# Characterization of Spray Modes and Factors Affecting the Ionization Efficiency of Paper Spray Ionization

**DOI:** 10.3389/fchem.2022.864184

**Published:** 2022-04-08

**Authors:** Thi Minh Hoa Nguyen, Woo-Young Song, Tae-Young Kim

**Affiliations:** School of Earth Sciences and Environmental Engineering, Gwangju Institute of Science and Technology, Gwangju, South Korea

**Keywords:** paper spray ionization, spray mode, ionization efficiency, mass spectrometry, hydrophobic paper

## Abstract

In this study, we systematically evaluated the factors affecting the ionization efficiency of paper spray ionization (PSI), such as electric field, solvent supply rate, and paper thickness and hydrophobicity. The observed paper spray plume was classified into three modes: single cone-jet, multi-jet, and rim-jet modes. With the increase in the spraying voltage, the spray plume appeared in order of single cone-jet, multi-jet, and rim-jet modes. The rim-jet mode exhibited the lowest standard deviation and high ionization efficiency among the three spray modes. The main parameter determining the spray mode was the charge density of the droplets generated by paper spray, which depends on the electric field and solvent supply rate. A thicker paper reduced the electric repulsion between the jets and lowered the threshold voltage to reach the rim-jet mode. Lowering the solvent supply rate caused mode transitions from the single cone-jet to the rim-jet, possibly due to the increased droplet charge density. The hydrophobic modification on a paper substrate led to a different ionization mechanism or electrostatic spray ionization at low applied voltages.

## Introduction

Ambient ionization mass spectrometry (MS) has emerged as a group of analytical techniques that directly analyze samples from their native environment with little or no sample preparation (Cooks et al., 2006; Lu et al., 2018; Kuo et al., 2019). Among a wide range of ambient ionization methods, paper spray ionization (PSI) has demonstrated significant potential for rapid analysis of biological, forensic, and environmental samples in complex matrices (Feider et al., 2019; Kuo et al., 2019). To improve sampling and concentration of analytes and/or enhance the diffusion and formation of ions, generic cellulose paper can be coated with various materials, such as silica (Zheng et al., 2015; Damon et al., 2016a), zirconia (Zheng et al., 2016), polystyrene (Wang et al., 2017), wax (Damon et al., 2016b), and carbon nanotubes (Han et al., 2015; Zheng et al., 2016). Several PSI studies exploited silanization to convert normal hydrophilic papers into hydrophobic ones, resulting in the more sensitive detection of target analytes (Damon et al., 2016a; Basuri et al., 2019). The success of PSI has also spurred the use of other cellulose-based materials such as parts of a plant (leaves, roots, stems, flowers, fruits, and seeds) (Liu et al., 2011), wooden toothpicks (Hu et al., 2011; Deng et al., 2017), and thread (Jackson et al., 2018) as substrates for electrospray ionization (ESI) MS.

The ionization process in a paper spray is governed by several experimental parameters. Espy et al. observed two distinguished spray modes in normal positive-mode PSI conditions (Espy et al., 2012). As observed in a conventional ESI mass spectrum, mode 1 at the beginning of the PSI process, where the solvent is rich, produces an unstable multi-jet spray with a broad droplet size distribution and protonated ions. In contrast, mode 2 occurred when most of the solvent was depleted, resulting in small droplets of size less than 1 μm with a nearly monodisperse distribution and radical ions during atmospheric pressure chemical ionization (APCI). Kim et al. reported another APCI mode in PSI under the conditions of a high flow rate of a nonpolar solvent and a high voltage of approximately 6 kV (Kim et al., 2017). This technique, named as paper spray chemical ionization, demonstrated sensitivity comparable to that of nano-ESI for low- and nonpolar compounds. Physical properties of paper, such as the angle of the paper tip, pore size, and thickness, and the selection of the spray solvent have significant impacts on the ionization efficiency of analytes during paper spray (Yang et al., 2012; Bills et al., 2018).

Paper spray shares some common features with electrospray in the mechanism of ion formation (Liu et al., 2010). Previous studies on electrospray performance classified the emission of fluid droplets at the capillary needle into several modes—cone-jet, multi-jet, and rim-jet—and demonstrated an increase in the number of emission points with an increase in the electric potential (Ryan et al., 2014; Kumar et al., 2018; Zheng et al., 2018). Electrospray is also sensitive to change in the electric field of an ion source. The electric field (*E*) generated at the ESI tip to a grounded counter electrode can be calculated by the following equation;

$$\;E=\;\frac{V}{A\cdot r\cdot \ln\left(\frac{4d}{r}\right)}$$ (1)

where *V* is the applied voltage, *r* is the emitter radius, *d* is the distance between the capillary tip and the ground electrode, and *A* is an empirical constant (Thompson et al., 2005). It can be observed that the physical parameters associated with the experimental setup directly affect the ESI performance. Ryan et al. demonstrated that the applied voltage has a linear relationship with the flow rate of the emitted spray for three electrospray modes or pulsation, cone-jet, and multi-jet modes (Ryan et al., 2014). In brief, the electric field at the capillary emitter directly influences the spray modes and subsequently the mass spectrometric signals in ESI.

Given the similarities between PSI and ESI, the paper tip in PSI can be regarded as the capillary emitter in ESI. Thus, adjusting the PSI physical parameters, such as the applied voltage and paper geometry, will cause a change in the electric field, thereby affecting the spray modes. Pioneering studies on the PSI mechanism have already reported the importance of the paper tip angle and the dependence of the spray jets on the solvent flow rate and applied voltage (Wang et al., 2010; Yang et al., 2012). However, the term “tip angle” may not be sufficient to fully describe the paper geometry because the paper tip is not of a cylindrical cone shape as that observed in the ESI emitter tip. The thickness of the paper is another parameter of paper geometry that should be considered as it affects the sharpness of the paper tip. Bills et al. demonstrated the effect of the paper thickness on the recovery and sensitivity of PSI; however, they did not investigate its possible impact on the spray modes (Bills et al., 2018).

Herein, we aim at supplementing the fundamental understanding of the underlying processes occurring during PSI through a systematic investigation of the effects of the electric field and solvent flow rate on the paper spray plume. To monitor changes in the spray plume by varying the PSI source parameters, the images of the paper spray were recorded using a digital camera through the side view. The effect of the electric field was examined considering the following three parameters: the applied voltage, paper thickness, and solvent supply rate. Finally, the interaction of the paper substrate with the solvent was examined through experiments comparing unmodified and hydrophobic papers.

## Materials and Methods

### Chemicals and Materials

Three papers used in this study—Whatman grade 40 filter paper, grade 3 MM chromatography paper, and grade 17 chromatography paper—were purchased from GE Healthcare (Buckinghamshire, United Kingdom). Antibiotics used in the experiments as analytes were HPLC-grade trimethoprim (purity >97.0%) purchased from FUJIFILM Wako Pure Chemical Corporation (Tokyo, Japan), and erythromycin from Sigma (St. Louis, MO, United States). Heavy-isotope-labeled trimethoprim (pyrimidine-4,5,6-^13^C_3_, 99%) in methanol (50 μg/ml) was obtained from Cambridge Isotope Laboratories (Tewksbury, MA, United States). All HPLC-grade organic solvents and formic acid were purchased from Thermo Fisher (St Wyman, MA, United States). Stock solutions of antibiotics were prepared at a concentration of 2 mg/ml in a water:methanol (1:1, v/v) solution and then stored at −20°C before use.

To synthesize the hydrophobic paper, papers were incubated in a desiccator with an open glass vial containing 80 μL of the trichloro (3,3,3-trifluoropropyl) silane reagent. The desiccator was connected to a vacuum pump (DOA-V750-AC, Gast, Benton Harbor, MI, United States), which was set to −0.5 bar. The silane reagent reacts with the hydroxyl group of cellulose, as reported by Damon et al. (2016a). The hydrophobicity of the paper was proportional to the exposure time. After the reaction, the modified paper was immediately washed with methanol to eliminate any unreacted reagent in the paper to stop the further reaction. We conducted this quenching step to enable the reproducibility of the synthesis reaction results. After the synthesis, the hydrophobic papers were treated similarly to the unmodified paper. Herein, the term hydrophobic paper refers to the paper exposed for 3 h, unless specified otherwise.

### PSI-MS

All types of paper were cut into an isosceles triangle with one apex at 30° and a height of 10 mm. The triangular papers were washed three times with methanol for 5 min in an ultrasonic cleaner, and were ready to use after solvent evaporation in a fume hood at room temperature. The mass spectrometric experiments were conducted using an LTQ Velos mass spectrometer (Thermo Scientific, San Jose, CA, United States). Optimization of collision energy and selecting product ions for each compound were performed using the LTQ Tune plus software. The data from other experiments were recorded in both full scan and selected reaction monitoring modes using the Xcalibur software with the MS/MS transition information provided in Supplementary Table S1. The triangular paper was held by a copper clip on a position-adjustable platform placed in front of the mass spectrometer inlet. A high voltage was applied through the copper clip. Thereafter, 1% formic acid in ethyl acetate as a spray solvent was continuously delivered by a solvent capillary connected to a syringe pump of the LTQ Velos spectrometer. Ethyl acetate was chosen as a common solvent for both hydrophilic and hydrophobic papers in PSI based on its substrate wettability, ionization efficiency, and solubility of the target analytes. In addition, 1% of formic acid was used as an additive to enhance the protonation of analytes. A Dino-lite digital microscope was set up to record all the spray plumes of PSI from either the top view or side view (Supplementary Figure S1). For each condition, four replicates were run.

### Effect of the Applied Voltage on the Spray Mode

Three types of paper were used to ionize the sample solution by applying a voltage of 1,500–6250 V, with increments of 250 V. The sample solution contained 10 μg/L trimethoprim, 50 μg/L erythromycin, and 10 μg/L heavy-isotope-labeled trimethoprim in the spray solvent (1% formic acid in ethyl acetate). This solution was supplied at a flow rate of 50 μL/min.

### Effect of the Flow Rate on the Spray Mode

The paper tip was loaded with 5 μL of the antibiotic mixture solution and dried under ambient conditions. The observed flow rates were 20, 30, 50, and 80 μL/min, and the applied voltage was 5000 V.

### Hydrophobic PSI

To make the silanized paper emitters with varying degrees of hydrophobicity, the exposure times to the silanization reagent were set as 2, 3, and 4 h. PSI-MS was performed using these three hydrophobic paper emitters and a normal hydrophilic paper emitter under a continuous supply of ethyl acetate solution containing trimethoprim (10 μg/L) and erythromycin (50 μg/L) at a flow rate of 50 μL/min. Thereafter, the PSI signal intensities of trimethoprim and erythromycin were measured.

Hydrophobic papers with an exposure of 3 h were utilized as the substrate to study the effect of distance and applied voltage on the PSI emitter. In this study, the term “distance” indicates the distance between the paper tip and MS inlet, and it was tested from 2 to 7 mm. The applied voltage varied from 2000 to 6750 V. The sample solution contained 10 μg/L trimethoprim, 50 μg/L erythromycin, and 10 μg/L heavy-isotope-labeled trimethoprim in the spray solvent. This solution was supplied at a flow rate of 50 μL/min. Four technical replicates were measured using a single paper substrate for each combination of applied voltage and distance.

## Results and Discussion

### Effect of the Electric Field on the Spray Mode

**FIGURE 1:**
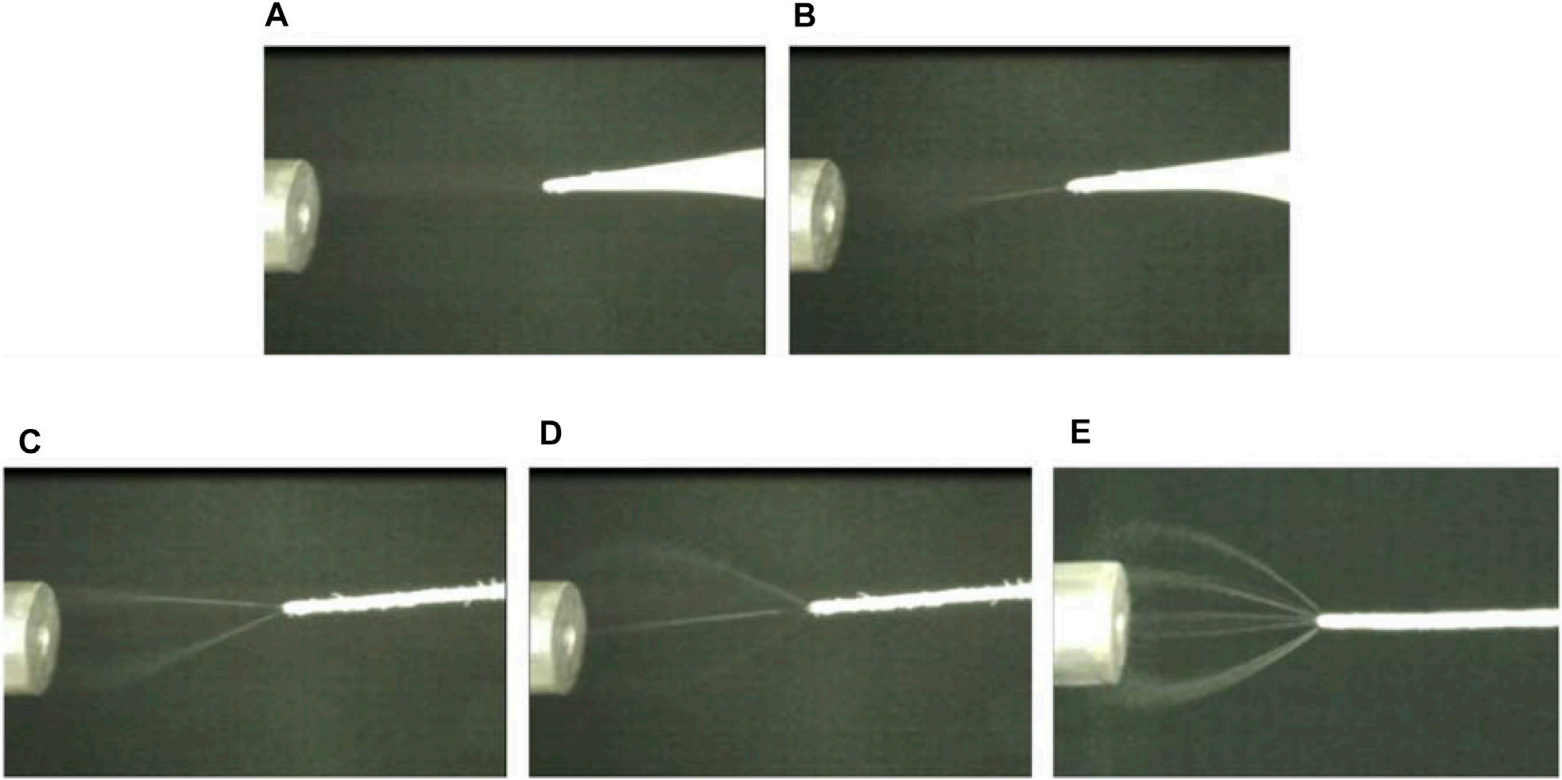
Images of the spray plumes obtained from the side of the paper with increasing applied voltage with a spray flow rate of 50 μL/min. **(A)** No visible spray jet, **(B)** single cone-jet mode, **(C)** multi-jet mode (two visible jets), **(D)** multi-jet mode (three visible jets), and **(E)** rim-jet mode.

The microscopic images recorded from the side view revealed the changes in the spray mode under different experimental conditions. In this study, the spray modes were defined by the number of visible jets; single-jet for only one visible spray jet, multi-jet for two, or three jets, rim-jet for more than three jets (Figure 1). The spray mode depends on the electric field that can be modified by varying the applied voltage. The spray mode changed from single-jet to multi-jet and then rim-jet as the applied voltage increased from 1,500 to 6250 V. We defined the onset voltage of the spray mode as the minimum applied voltage that initiates a specific spray mode. The average onset voltages of four replicate experiments for single-, multi-, and rim-jet modes were 2.3, 3.7, and 5.2 kV, respectively. The formation of multiple jets from a single paper tip can be attributed to the high electric field generated at the fibrous microstructure of the paper tip. Previous studies on the electrospray droplets through the capillary needle reported an increase in the number of emission points with the increasing electric potential (Ryan et al., 2014; Kumar et al., 2018; Zheng et al., 2018).

It is noteworthy that when the spray mode transits from the single cone-jet to multi-jet mode, the second and third jets can be observed only from the side view (Supplementary Figure S2). The new extra jets were formed far from the former since spray jets consisting of charged droplets with the same polarity repel each other. The formation of the spray jets along the axis orthogonal to the triangular paper surface appeared to be the origin of the off-axis paper spray jets shown in the high-resolution image observed from the top view by Espy et al. (2012). With even higher applied voltages, the rim-jet mode consisting of a collection of spray jets from all the directions around the paper apex was observed.

**FIGURE 2:**
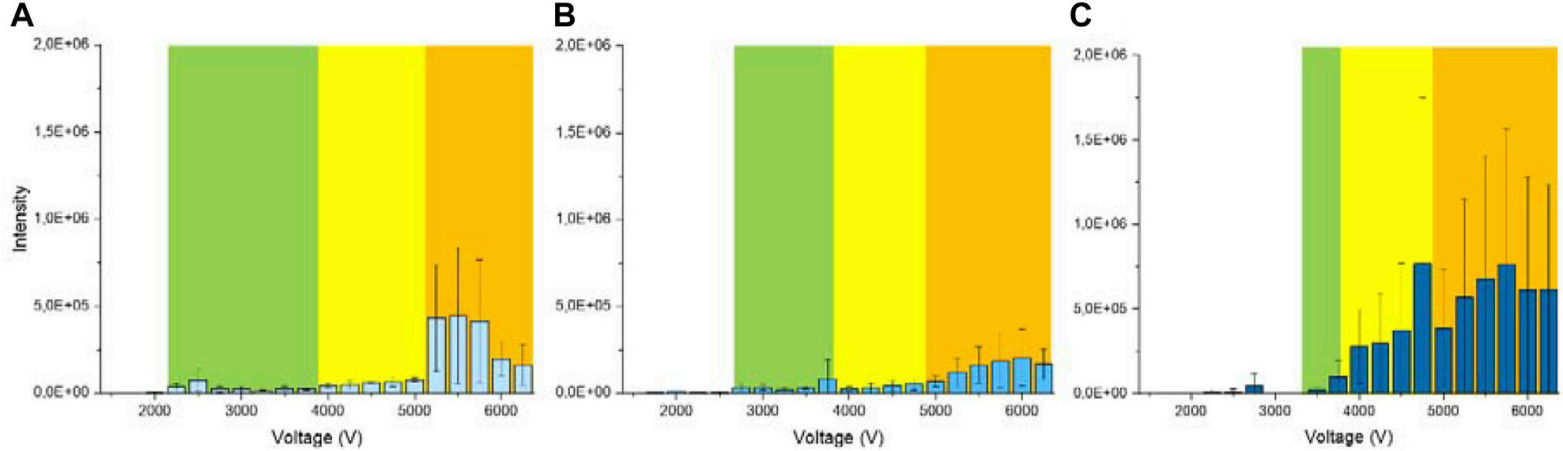
PSI MS signal intensity of 50 μg/ml trimethoprim loaded onto the **(A)** Whatman grade 40 filter paper, **(B)** grade 3 MM chromatography paper, and **(C)** grade 17 chromatography paper with varying spray voltages. The background colors indicate the spray modes: green, yellow, and orange corresponding to the single cone-jet, multi-jet, and rim-jet modes, respectively.

The signal intensity increased as the spray mode changed from the single cone-jet to rim-jet modes with the increasing applied voltage (Figure 2). The observed tendency of the MS signal intensity depending on the spray voltage is in agreement with the results obtained by Wang et al. (2010). The stronger electric field could result in droplets of smaller diameters, leading to more efficient solvent evaporation and gas-phase ion formation. Hollerbach et al. observed a significant decrease in droplet size of nano-ESI with the increasing spray voltage (Hollerbach et al., 2018), which could be explained by the fact that a strong electric field is advantageous to overcome the surface tension of the solvent. PSI can generate multiple jets because of the fibrous structure of paper. Increasing the strength of electric field leads to a greater number of spray jets from different microscopic sites of the paper tip, which can further reduce the droplet diameter since the solvent flow rate per jet decreases at a given solvent flow rate.

### Impact of Paper Thickness on the Onset Voltage of the Spray Mode

**FIGURE 3:**
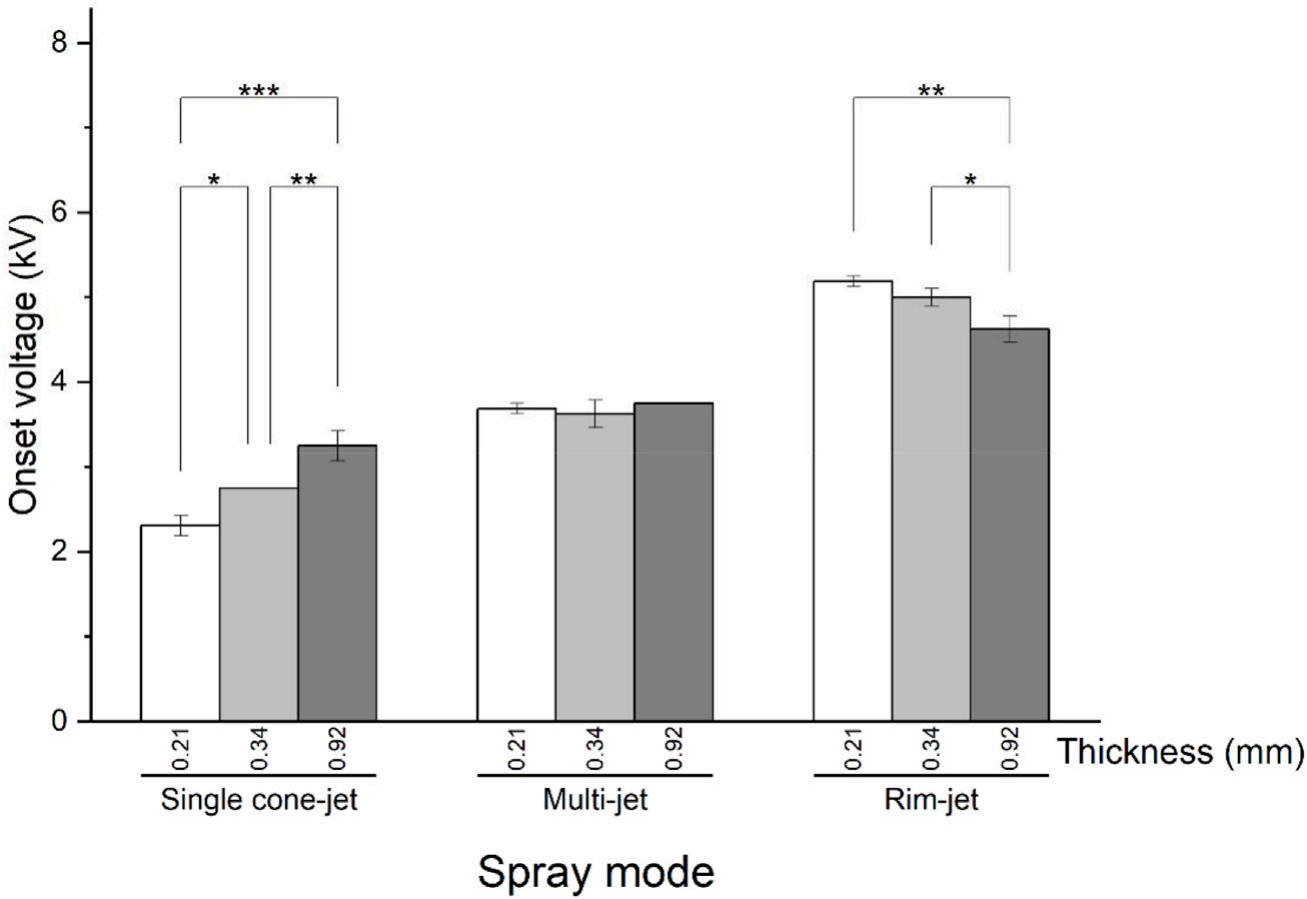
Variations in the onset voltages for three spray modes with different paper thicknesses: *p < 0.05, **p < 0.01, ***p < 0.001.

Figure 3 shows the variations in the onset voltage with respect to paper thickness. In the single cone-jet mode, the onset voltage significantly increased with the increasing paper thickness, which can be explained using Eq. 1 if the paper thickness is regarded as the emitter radius in ESI and the thickness is sufficiently small. In contrast, in the rim-jet mode, the onset voltage decreased with the increase in the paper thickness. Interestingly, the multi-jet mode did not exhibit any significant difference in the onset voltage among the papers with different thicknesses.

The tendencies of the onset voltage with respect to the paper thickness in the rim-jet and multi-jet modes can be interpreted based on the study by Kumar et al. (2018) on the performance of multiple electrospray emitters. In their study, the measured electric field at the emitters in a linear array with the fixed applied voltage increased with the increasing distance between the emitters. This is because the electrical repulsion between the sprays with the same polarity decreases with an increasing distance between the emitters. In our experiment, the spray jets in the rim-jet and multi-jet modes can be considered as the emitters arranged adjacent to each other. The average distances between the adjacent jets in the rim-jet mode were measured as 0.10 and 0.29 mm for paper tips with thickness of 0.34 and 0.92 mm, respectively (Supplementary Figure S3). The larger space between the spray jets in the thicker paper reduces the repulsive force between the jets in the rim-jet mode. Thus, there was a significant reduction in the onset voltage for the thicker paper. In contrast, the multi-jet mode has only two or three jets for which the distance between them in the paper tip is large enough to not cause any significant electrical repulsion. In summary, the paper thickness indicates the size of the emitter in the single cone-jet mode, while it provides a space for multiple jets to co-exist in the other two modes.

### Influence of Solvent Supply Rate on the Spray Mode

**FIGURE 4:**
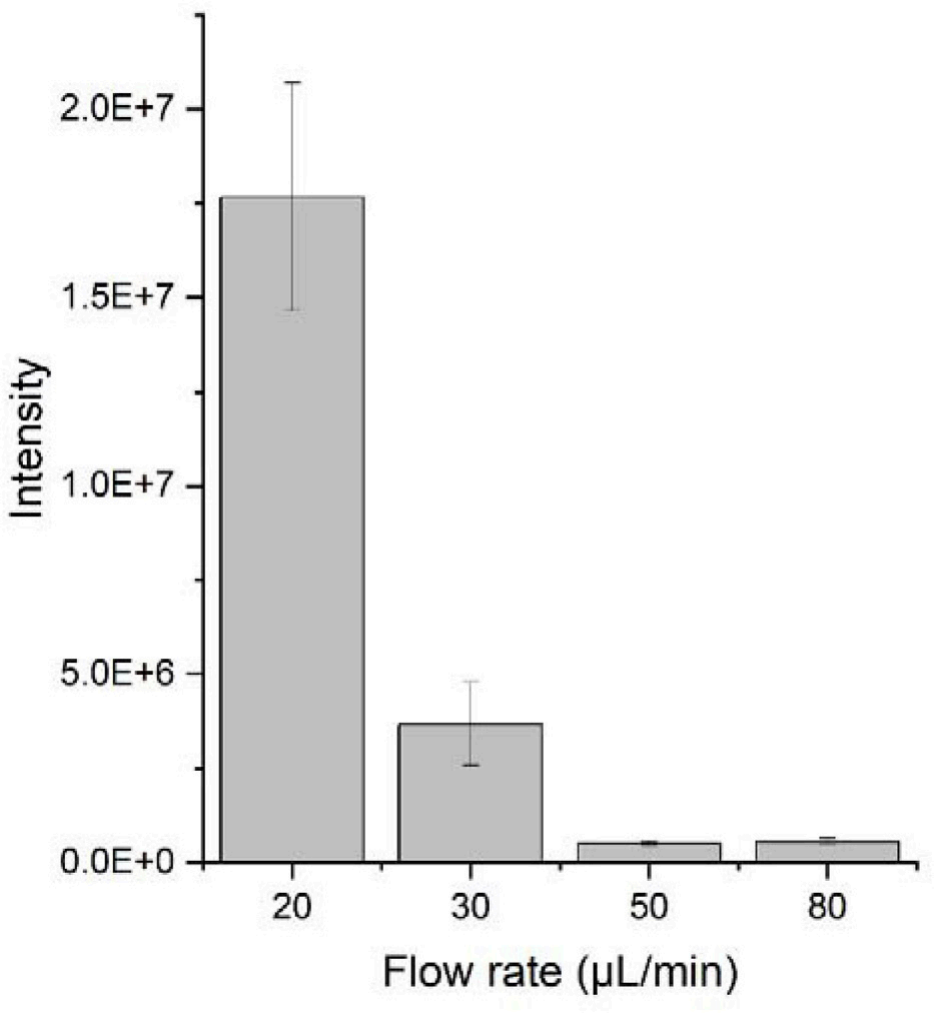
PSI MS signal intensity of 50 μg/ml trimethoprim analyzed at the spray voltage of 5 kV and solvent flow rates varying from 20 to 80 μL/min.

In a typical PSI experiment, the spray mode changes as the solvent is depleted over the time when a fixed amount of elution solvent is loaded onto the paper (Espy et al., 2012). In this study, we monitored the change in the spray mode while the rate of solvent supply was controlled, as demonstrated by Vandergrift et al. (2018). As the solvent flow varied from 20 to 80 μL/min at a constant spray voltage, the spray plume shifted from the rim-jet to multi-jet and then single cone-jet mode (Supplementary Figure S4). These results imply that the spray mode also depends on the solvent supply rate or the amount of solvent available at the paper tip. In other words, in a fixed voltage applied to the paper, a modification in the solvent flow rate affects the charge density at the paper tip. The higher charge density at the paper tip with the lower solvent supply rate prefers the rim-jet mode to the single cone-jet mode because highly charged droplets repel each other strongly. Figure 4 shows a significant decrease in the PSI MS signal intensity of an antibiotic with an increase in the solvent flow rate, which cannot be explained by the dilution of the sample in a large volume of solvent because the signal reduction trend does not match the dilution factors. Espy et al. demonstrated that a solvent-deficient condition in PSI results in fine droplets, leading to a higher signal intensity because of an enhanced evaporation efficiency (Espy et al., 2012). Thus, the higher signal intensity at the lower solvent flow rate can be attributed to the reduced droplet size.

Based on these experiments, we demonstrated that the charge density of the liquid at the paper tip, which is affected by both the electric field and the solvent flow rate, is the key factor to determine the spray mode in PSI. Lowering the solvent supply rate or increasing the electric field can increase the charge density of the liquid on the paper tip. The higher charge density of the liquid at the paper tip tends to spray in more jets.

### Effect of the Properties of the Paper Surface on the Spray Mode

**FIGURE 5:**
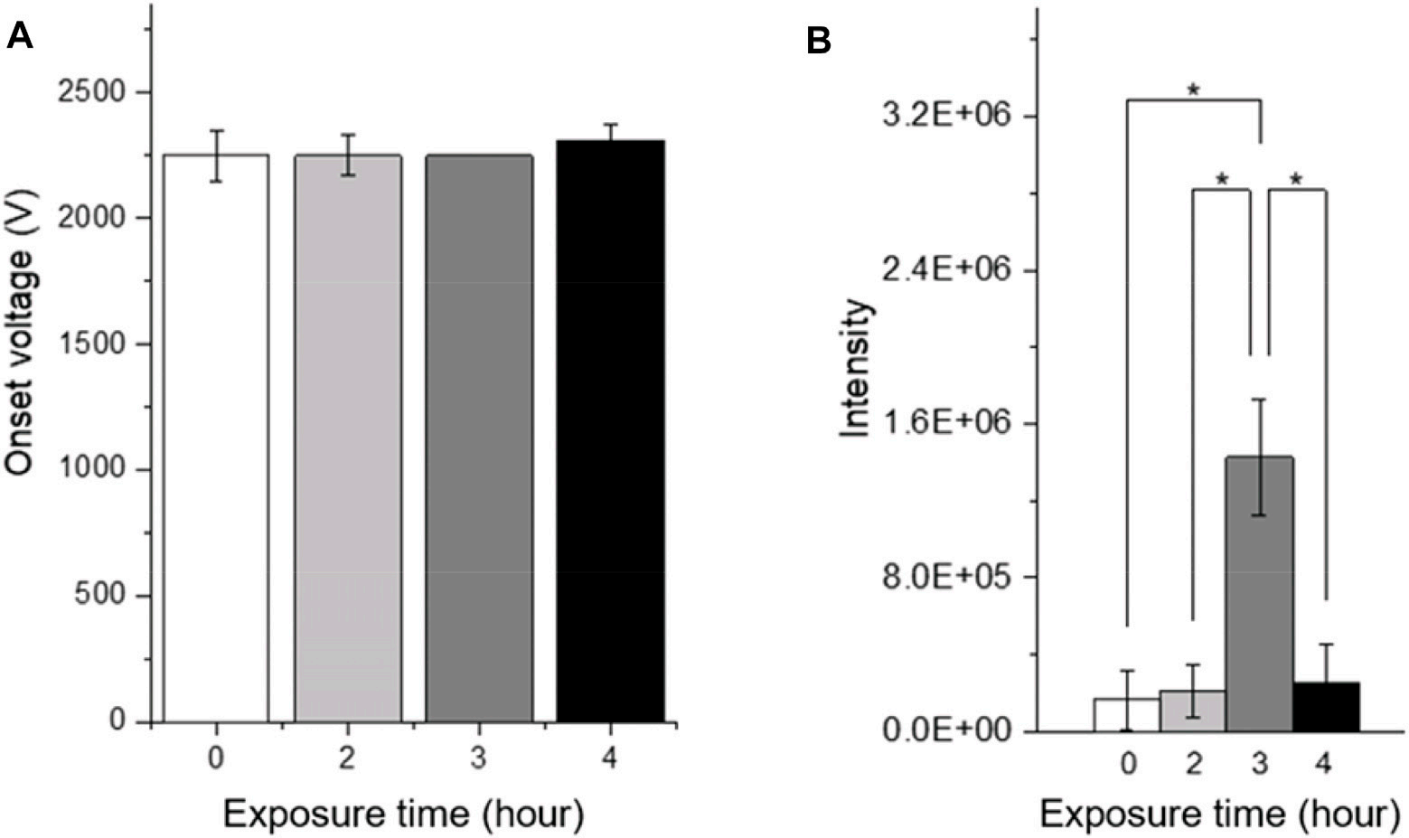
**(A)** Onset voltage of the single cone-jet and **(B)** signal intensity of 50 μg/ml trimethoprim using hydrophobic papers with different exposure time to the silanization reagent.

The hydrophobicity of the paper is another key factor affecting the ionization efficiency of PSI. We examined the change in the PSI sensitivity of hydrophobic compounds as a function of hydrophobicity of the paper, which was differentiated by varying the time of exposure to silanization reagent from 2 to 4 h. The paper exposed to the silanization reagent for 3 h resulted in approximately 8 and 3 times higher signal intensities of trimethoprim and erythromycin, respectively than those in a normal hydrophilic paper (Figure 5). In contrast, no significant differences in signal intensities of the antibiotics were observed between the papers exposed to the silanization reagent for 2 and 4 h and the normal hydrophilic paper.

The enhancement of signal intensity using paper with a specific exposure time (3 h) is consistent with the results reported by Damon et al. (2019). They found that the signal intensity in PSI is maximized when the surface energy of the substrate matches the surface tension of the wetting solvent. An extremely hydrophobic paper with the surface energy of the substrate lower than the surface tension of the solvent leads to a low signal intensity due to the unstable Taylor cone and reduced rate of desorption of droplets from the paper tip.

**FIGURE 6:**
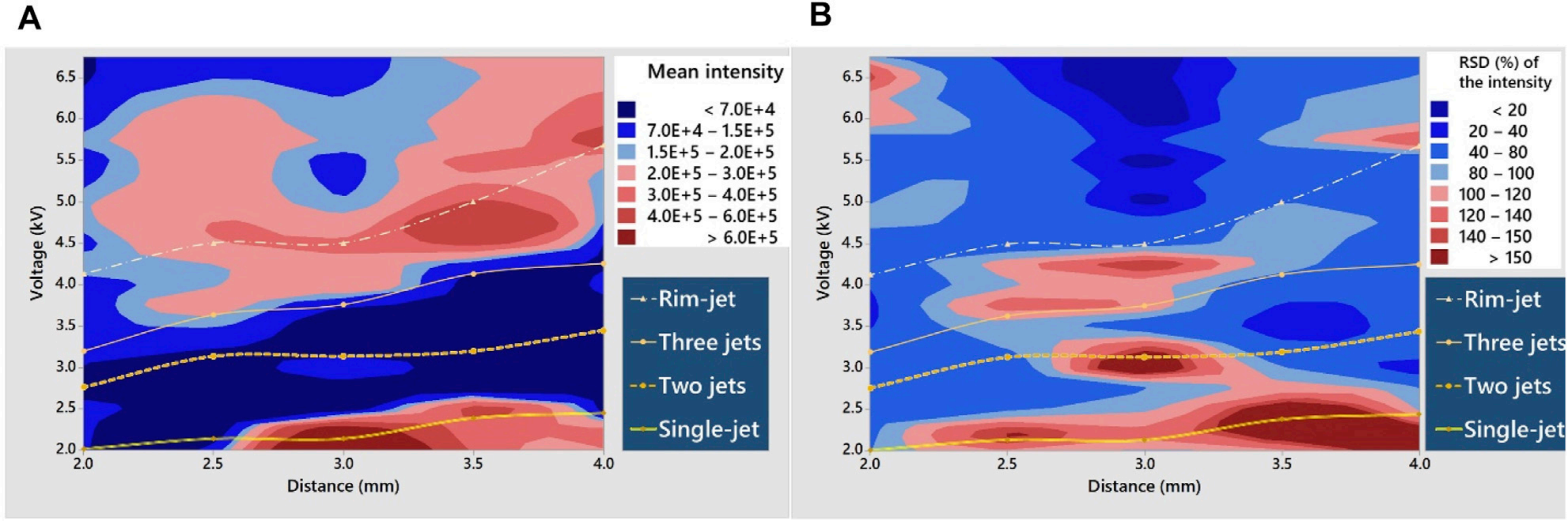
Contour plots of the **(A)** PSI MS signal intensity and **(B)** its RSD of 50 μg/ml trimethoprim at a solvent flow rate of 50 μL/min with various combinations of the applied voltage and the distance between the paper tip and the MS inlet. The lines indicate the average onset voltages of different spray modes.

Figure 6A shows the MS intensities of trimethoprim loaded on the hydrophobic paper under various experimental conditions. High signal intensity was observed in the area around the line of the onset voltage of the single cone-jet mode. The signal intensity was also high in the areas with the three-jet and rim-jet modes, but it was low in the area with the two-jet mode. The PSI MS signal intensities of erythromycin exhibited a similar trend to those of trimethoprim (Supplementary Figure S5). These results are consistent with the pattern of signal intensities depending on the spray mode observed in the normal hydrophilic paper, except for the area around the line of the onset voltage of the single cone-jet mode. At voltages lower than the onset voltage of the single cone-jet mode, the normal hydrophilic papers exhibited nearly zero signal intensities (Figure 2). On the contrary, the hydrophobic paper exhibited a high signal intensity under the same conditions, although no visible jets were observed. This observation implies that the ions on the hydrophobic paper were formed by a different mechanism instead of ESI.

The formation of a stable Taylor cone is a prerequisite for ESI. However, the Taylor cone formation is hindered at voltages lower than the onset voltage in PSI. Damon et al. suggested that electrostatic spray ionization can occur on a hydrophobic paper in PSI (Damon et al., 2019). For a hydrophobic paper, capacitive charging occurs in a solvent droplet on the paper tip, inducing electrostatic force along the axis connecting the paper tip and the MS orifice. An imbalance between the electrostatic force and the surface tension of the droplet results in macroscopic oscillation of the droplet on the paper tip. During the oscillation, the analytes with a sufficiently high kinetic energy that break through the droplet surface can be ionized. In contrast, the oscillating droplet is not formed on a normal hydrophilic paper owing to its high solvent wettability, resulting in no electrostatic spray ionization. In summary, the differential signal intensity between hydrophobic and hydrophilic papers at low voltage PSI conditions can be ascribed to their difference in solvent wettability.

Figure 6B shows the relative standard deviations of the signal intensity of trimethoprim. The standard deviations were the lowest at the rim-jet mode and the highest at the single cone-jet mode. The variations in the standard deviations of the signal intensity among the different spray modes can be explained by considering the microstructure of the paper. The microfiber structure of the paper can act as an array of multiple emitters for PSI. Kelly et al. demonstrated that an array of multiple emitters exhibited higher ESI signal stability owing to a relatively uniform electric field compared with a single emitter (Kelly et al., 2008).

However, unlike a hydrophobic paper, the relative standard deviation of the trimethoprim signal intensity at rim-jet mode was significantly larger in a normal hydrophilic paper (Figure 2). Jackson et al. pointed out that the interfacial tension between the solvent and the substrate is a major factor of electrospray (Jackson et al., 2017), the interfacial tension between the solvent and the substrate of a hydrophobic paper is weaker than that of a normal hydrophilic paper due to a lower surface energy of a hydrophobic paper. The lower interfacial tension of a hydrophobic paper compared with a normal hydrophilic paper was also demonstrated by the greater contact angle of a water droplet on a hydrophobic paper compared with a normal hydrophilic paper (Supplementary Figure S6). The lower interfacial tension of a hydrophobic paper causes the longer electrohydrodynamic length of Taylor cone, resulting in droplets of smaller diameters (Gañán-Calvo et al., 2013) (Supplementary Figure S7). Since the smaller droplet size is more favorable for complete droplet evaporation, the fine droplet formation can lead to a reduced relative standard deviation and greater sensitivity for the PSI signal. Thus, the higher PSI signal stability and sensitivity of a hydrophobic paper compared with a normal hydrophilic paper can be explained by the formation of smaller droplet. In summary, different patterns in the signal intensity and stability of each spray mode observed between hydrophobic and hydrophilic papers can be ascribed to the differential surface energy of the two types of papers.

The spray mechanism of PSI described in this study could be applied to other spray ionization methods based on a porous solid substrate. The spray conditions including the applied voltage, distance between the tip and MS orifice, substrate thickness, solvent flow rate, and surface energy are shared among various spray ionization methods utilizing solid substrates and have significant impacts on the sensitivity and stability of the MS signals.

## Conclusion

We systematically investigated the characteristics of three spray modes, including single cone-jet, multi-jet, and rim-jet modes of normal and hydrophobic PSI. The spray mode was influenced by the charge density of the liquid surface at the paper tip, which depends on the solvent supply rate, applied voltage, and paper thickness. Increasing the solvent supply rate decreased the signal intensity and increased the onset voltage. With the increase in paper thickness, the PSI onset voltage of the single cone-jet mode increased, but that of the rim-jet mode decreased. The hydrophobic modification on the paper substrate reduced the surface energy of the paper, leading to dominant electrostatic ionization at low applied voltages. A significant enhancement in the PSI sensitivity was observed when the surface hydrophobicity matched the solvent surface tension. The relationship between the spray modes of PSI and various spraying parameters demonstrated by this work can be of significant importance for the improvement and optimization of PSI.

## Data Availability

The original contributions presented in the study are included in the article/Supplementary Material, further inquiries can be directed to the corresponding author.
