# Supplementary material for: Characterization of Spray Modes and Factors Affecting the Ionization Efficiency of Paper Spray Ionization
Source: Front Chem. 2022 Apr 8;10:864184. doi: 10.3389/fchem.2022.864184 (PMC9024139; doi:10.3389/fchem.2022.864184)
Supplement: Supplementary file 1 [file DataSheet1.docx]

Supplementary Material

**Table of contents**

**Supplementary Table 1.** Selected reaction monitoring conditions for all antibiotics.

**Supplementary Figure 1.** Microscope setup for imaging of paper spray. (A) top and (B) side views.

**Supplementary Figure 2.** Images of the multi-jet spray mode observed from the side and top of a paper at the same condition.

**Supplementary Figure 3.** Images of spray jets using paper tips with different thickness of (A) 0.34 and (B) 0.92 mm.

**Supplementary Figure 4.** Variation of spraying modes by changing the flow rate from (A1 and A2) 20, (B1 and B2) 30, (C1 and C2) 50, to (D1 and D2) 80 μL/min. The spray voltage was 4000 V for A1 to D1 and 5000 V for A2 to D2.

**Supplementary Figure 5.** Contour plots of the (A) PSI MS signal intensity and (B) its RSD of 50 μg/mL erythromycin at a solvent flow rate of 50 μL/min with various combinations of the applied voltage and the distance between the paper tip and the MS inlet. The lines indicate the average onset voltages of different spray modes.

**Supplementary Figure 6.** Image of water droplet on (A) unmodified paper and (B-E) hydrophobic papers with silanization times of 1-4 h, respectively.

**Supplementary Figure 7.** Schematic of Taylor cone of PSI using (A) a normal hydrophilic paper and (B) a hydrophobic paper.

# Supplementary Table 1. Selected reaction monitoring conditions for all antibiotics

| Analyte | m/z of Precursor ion ([M+H]^+^) | Normalized collision energy | Quantitative product ion |
| --- | --- | --- | --- |
| Erythromycin | 734.30 | 22 | 576.24 |
| Trimethoprim | 291.15 | 30 | 230.08 |
| Trimethoprim-^13^C_3_ | 294.15 | 30 | 233.10 |

*
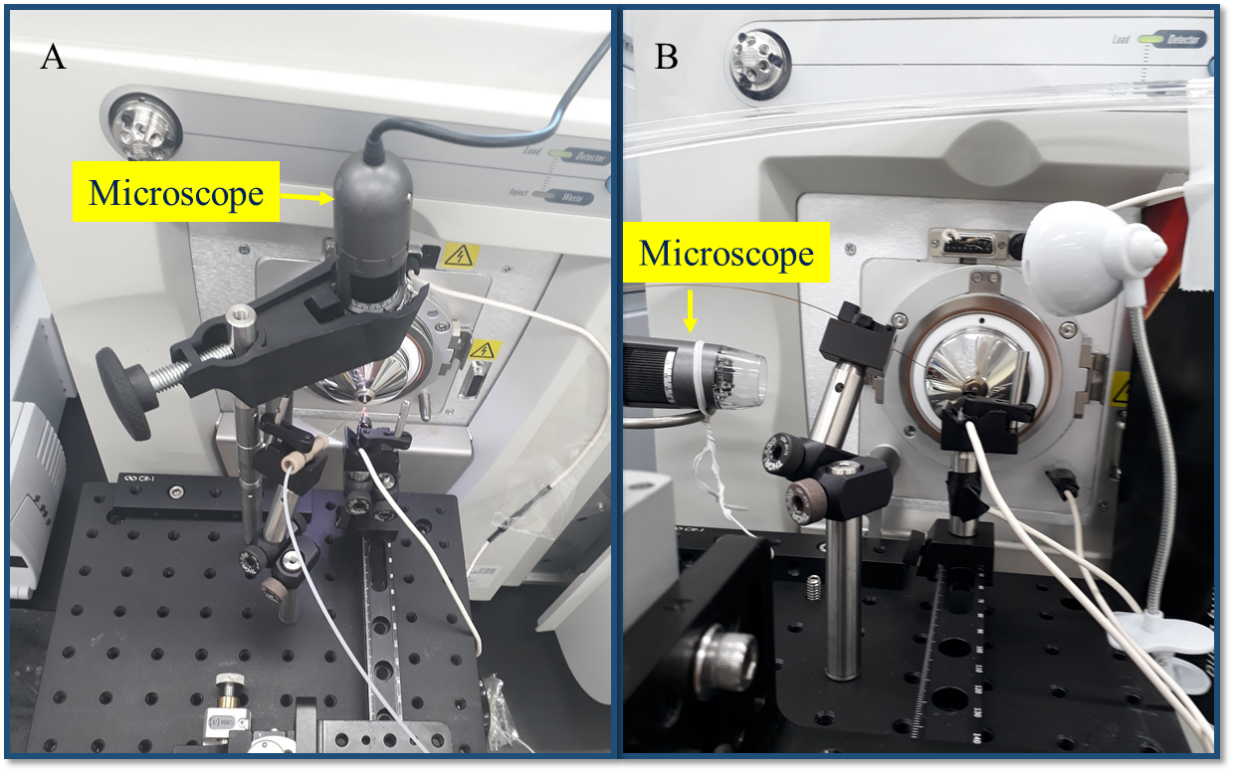
*

Supplementary Figure 1. Microscope setup for imaging of paper spray. (A) top and (B) side views.


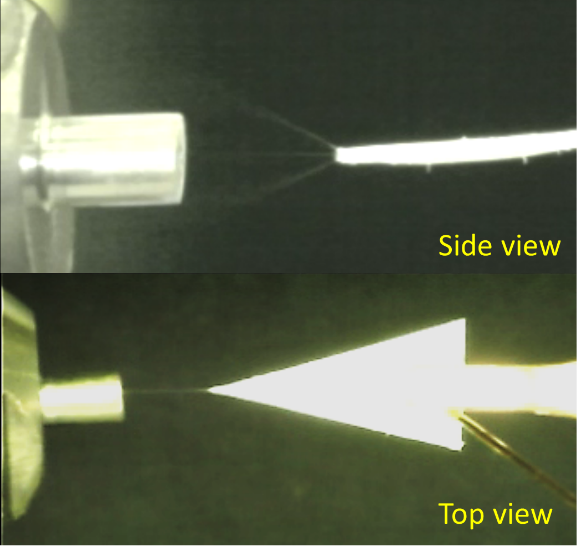


Supplementary Figure 2. Images of the multi-jet spray mode observed from the side and top of a paper at the same condition.

**
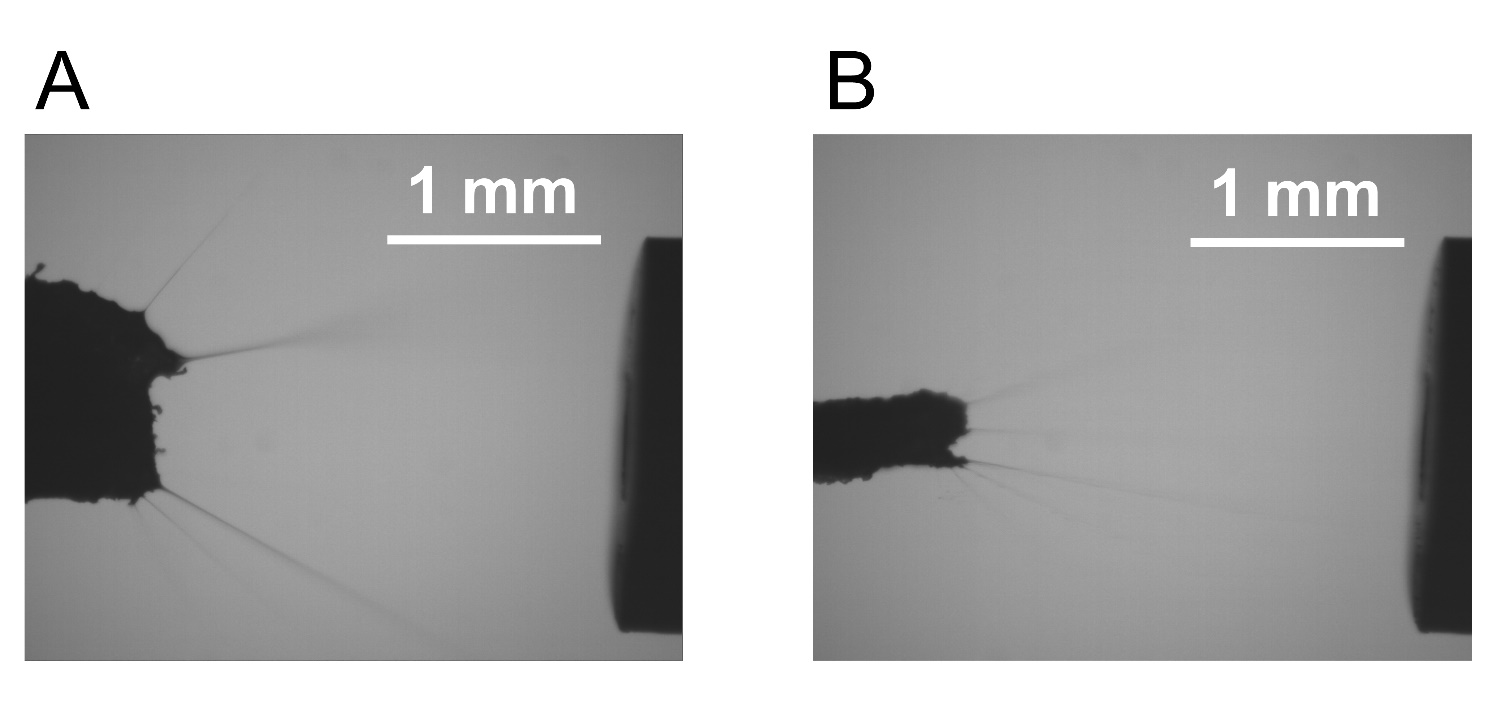
**

Supplementary Figure 3. Images of spray jets using paper tips with different thickness of (A) 0.92 and (B) 0.34 mm.


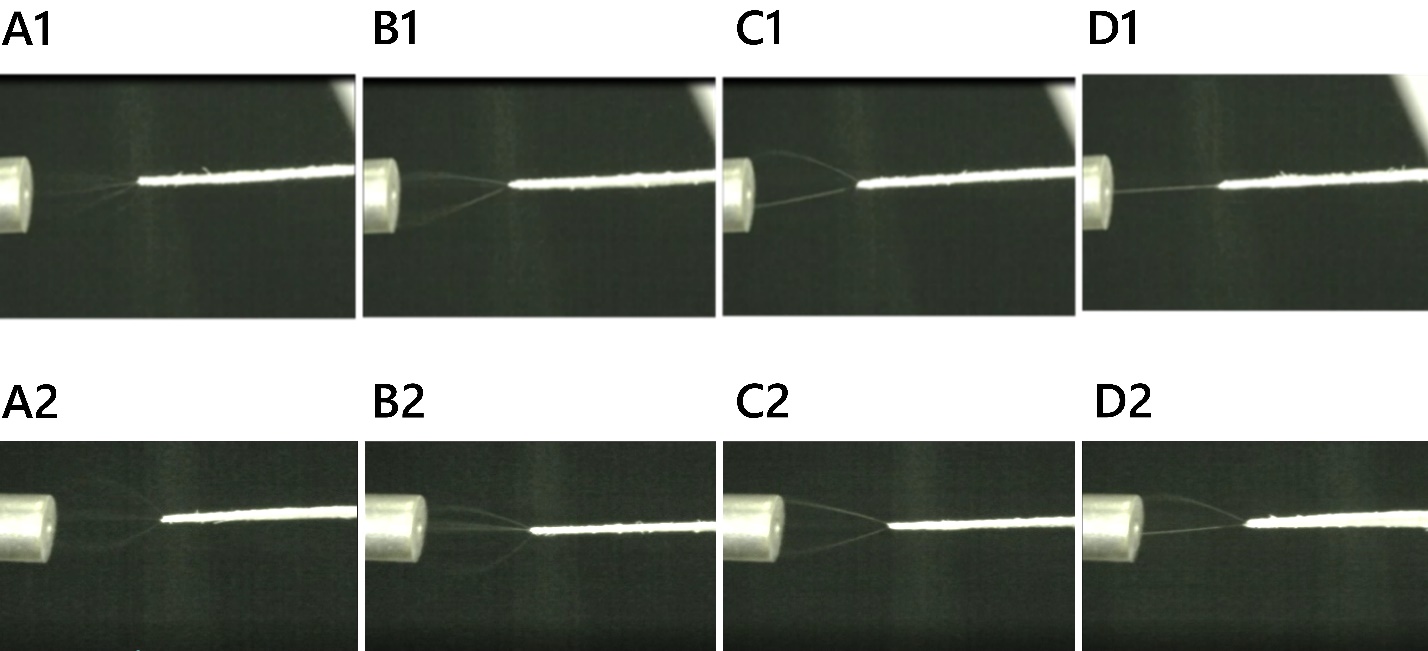


Supplementary Figure 4. Variation of spraying modes by changing the flow rate from (A1 and A2) 20, (B1 and B2) 30, (C1 and C2) 50, to (D1 and D2) 80 μL/min. The spray voltage was 4000 V A1 to D1 and 5000 V for A2 to D2.

*
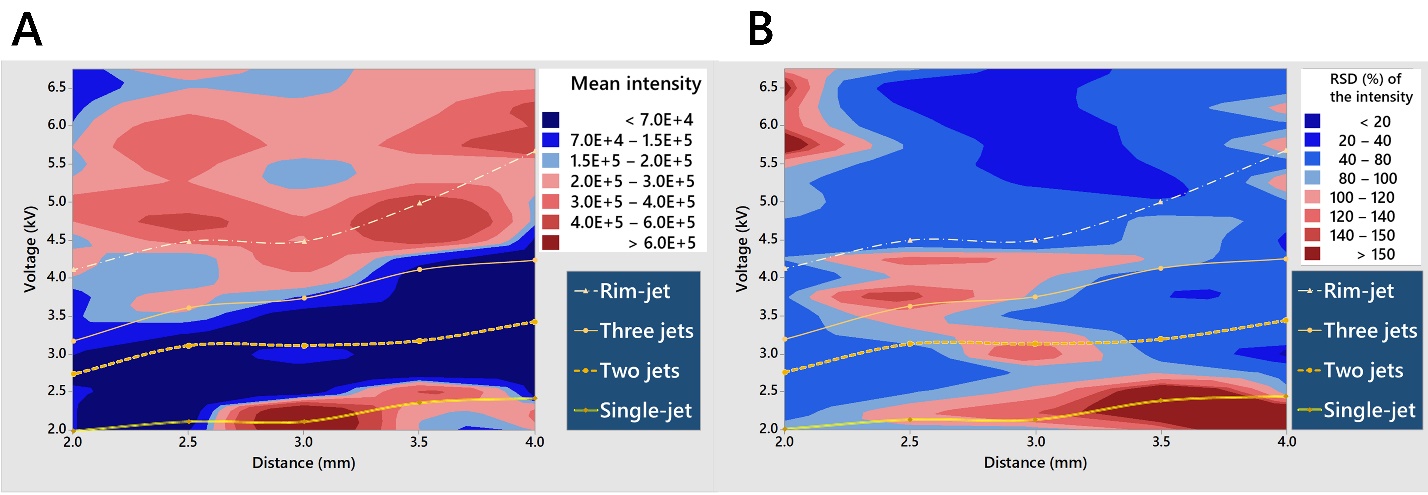
*

Supplementary Figure 5. Contour plots of the (A) PSI MS signal intensity and (B) its RSD of 50 μg/mL erythromycin at a solvent flow rate of 50 μL/min with various combinations of the applied voltage and the distance between the paper tip and the MS inlet. The lines indicate the average onset voltages of different spray modes.

**
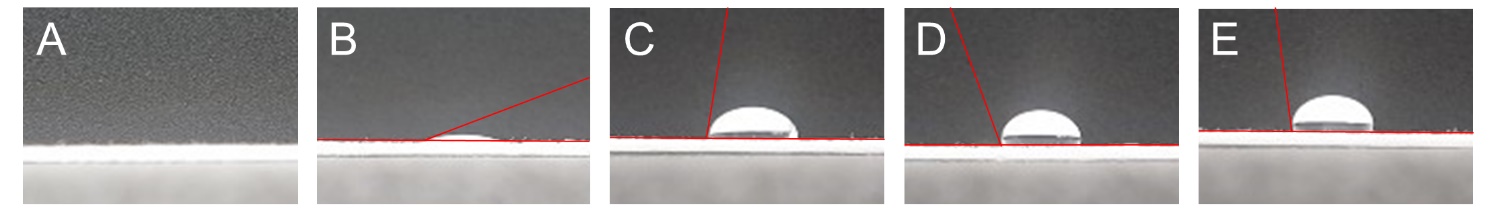
**

Supplementary Figure 6. Images of water droplet on (A) unmodified paper and (B-E) hydrophobic papers with silanization times of 1-4 h, respectively.


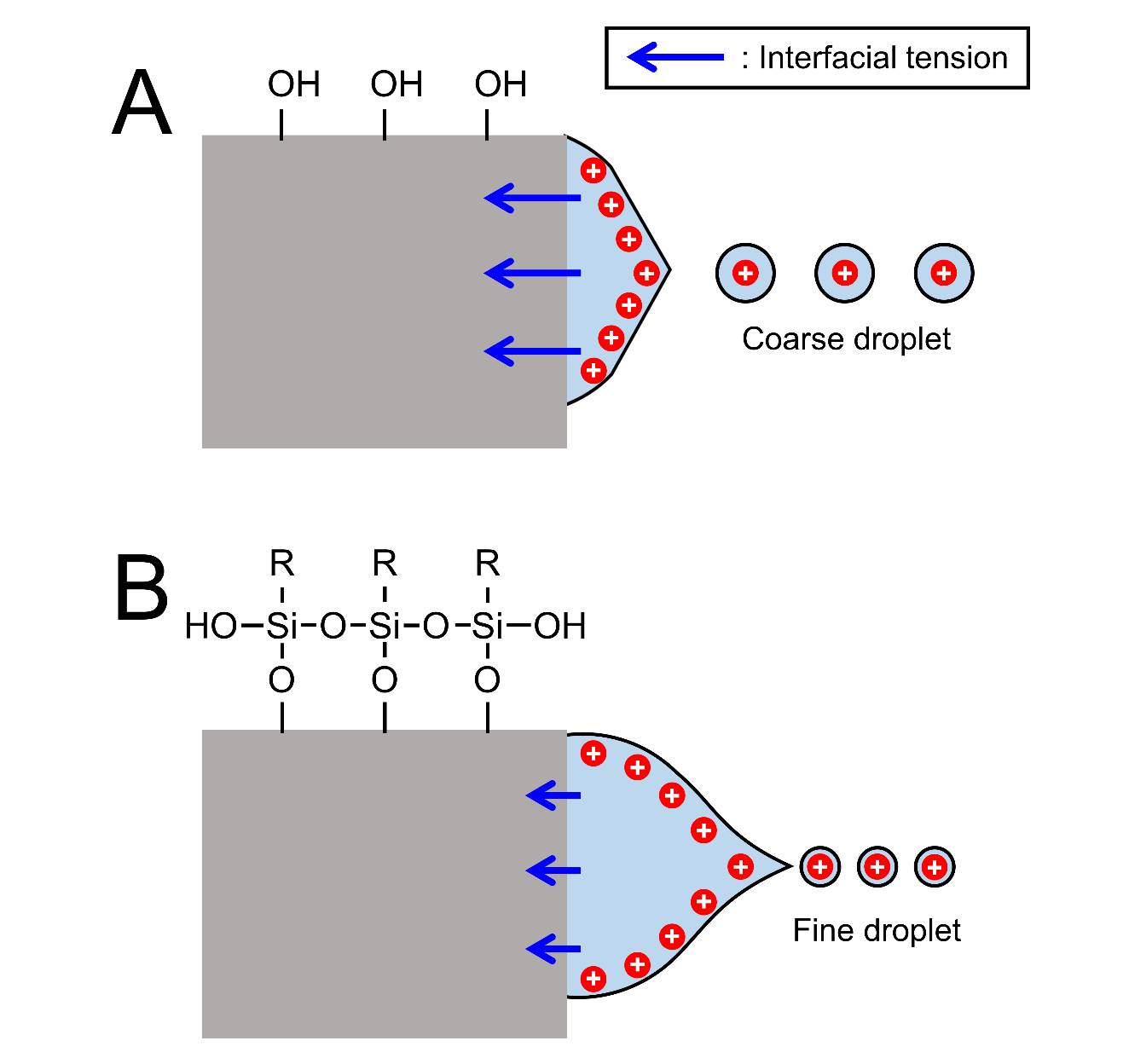


Supplementary Figure 7. Schematic of Taylor cone of PSI using (A) a normal hydrophilic paper and (B) a hydrophobic paper.
